# Environmental Enrichment Upregulates Striatal Synaptic Vesicle-Associated Proteins and Improves Motor Function

**DOI:** 10.3389/fneur.2018.00465

**Published:** 2018-07-16

**Authors:** Suk-Young Song, Minji Chae, Ji Hea Yu, Min Young Lee, Soonil Pyo, Yoon-Kyum Shin, Ahreum Baek, Jung-Won Park, Eun Sook Park, Ja Young Choi, Sung-Rae Cho

**Affiliations:** ^1^Department and Research Institute of Rehabilitation Medicine, Yonsei University College of Medicine, Seoul, South Korea; ^2^Graduate Program of NanoScience and Technology, Yonsei University, Seoul, South Korea; ^3^Rehabilitation Institute of Neuromuscular Disease, Yonsei University College of Medicine, Seoul, South Korea; ^4^Brain Korea 21 PLUS Project for Medical Science, Yonsei University, Seoul, South Korea; ^5^Department of Rehabilitation Medicine, Yonsei University Wonju College of Medicine, Wonju, South Korea; ^6^Department of Medicine, Yonsei University College of Medicine, Seoul, South Korea; ^7^Department of Rehabilitation Medicine, Eulji University School of Medicine, Daejeon, South Korea

**Keywords:** enriched environment, synaptic plasticity, synaptic vesicle, transport, exocytosis

## Abstract

Environmental enrichment (EE) is a therapeutic paradigm that consists of complex combinations of physical, cognitive, and social stimuli. The mechanisms underlying EE-mediated synaptic plasticity have yet to be fully elucidated. In this study, we investigated the effects of EE on synaptic vesicle-associated proteins and whether the expression of these proteins is related to behavioral outcomes. A total of 44 CD-1® (ICR) mice aged 6 weeks were randomly assigned to either standard cages or EE (*N* = 22 each). Rotarod and ladder walking tests were then performed to evaluate motor function. To identify the molecular mechanisms underlying the effects of EE, we assessed differentially expressed proteins (DEPs) in the striatum by proteomic analysis. Quantitative real-time polymerase chain reaction (qRT-PCR), western blot, and immunohistochemistry were conducted to validate the expressions of these proteins. In the behavioral assessment, EE significantly enhanced performance on the rotarod and ladder walking tests. A total of 116 DEPs (54 upregulated and 62 downregulated proteins) were identified in mice exposed to EE. Gene ontology (GO) analysis demonstrated that the upregulated proteins in EE mice were primarily related to biological processes of synaptic vesicle transport and exocytosis. The GO terms for these biological processes commonly included Synaptic vesicle glycoprotein 2B (SV2B), Rabphilin-3A, and Piccolo. The qRT-PCR and western blot analyses revealed that EE increased the expression of SV2B, Rabphilin-3A and Piccolo in the striatum compared to the control group. Immunohistochemistry showed that the density of Piccolo in the vicinity of the subventricular zone was significantly increased in the EE mice compared with control mice. In conclusion, EE upregulates proteins associated with synaptic vesicle transport and exocytosis such as SV2B, Rabphilin-3A and Piccolo in the striatum. These upregulated proteins may be responsible for locomotor performance improvement, as shown in rotarod and ladder walking tests. Elucidation of these changes in synaptic protein expression provides new insights into the mechanism and potential role of EE.

## Introduction

Environmental enrichment (EE) has the potential to elicit neurorestorative effects using complex combinations of physical, cognitive, and social stimuli (1). For laboratory animals, EE refers to group housing in large cages filled with various toys and tunnels, as well as equipment for voluntary exercise such as running wheels (2). EE can induce brain plasticity and enhance motor and cognitive function in the animal brain through biochemical and morphological changes such as neurogenesis, axonal sprouting, and dendritic branching (3–5)

In our previous studies, we conducted analyses of gene expression patterns to identify the effects of long-term exposure to EE. We observed alterations in levels of synaptic activity regulating genes including dopamine transporters (DAT) (6, 7). Genes associated with presynaptic neurotransmitter transporters such as DAT were down-regulated, suggesting that EE elicits efficient neurotransmitter reuptake and presynaptic plasticity. Synapses are important for constructing neural circuits and allowing electrical and chemical signals throughout the brain (8). Synaptic plasticity involves changes to these molecular components, which are present at synapses, and the efficiency with which synapses can communicate (8).

Previous studies have explored synaptic changes after EE, focusing on dendritic spines in synapses. Increased EE-induced spine density has been noted in the cortex and hippocampus (9, 10). EE increased synaptophysin, a major synaptic vesicle glycoprotein, as well as PSD95 in multiple brain areas, including the cortex, hippocampus, thalamus, and hypothalamus (11). As EE increases dendritic spines, synaptic molecules also undergo changes in expression. Synaptophysin is an integral membrane protein in synaptic vesicles (12, 13). Changes in levels of synaptophysin presumably reflect changes in synaptic vesicles, and it thus follows that such changes indicate synaptic plasticity.

In addition, synaptic changes after EE have been investigated especially in the cortex and hippocampus. EE modulates expression of the AMPA receptor and NMDA-R subunit in the cortex and hippocampus. EE upregulates cyclic adenosine monophosphate (cAMP) response element-binding protein (CREB) and protein kinase C (PKC) in the hippocampus and cortex (14). EE results in altered expression of hippocampal proteins that are associated with neuronal signaling and morphological changes. Accordingly, a broad range of mRNA and corresponding proteins (e.g., GTPase RhoA, PSD-95, actin binding proteins) show altered expression following EE (15). These changes are linked to synaptic plasticity-related processes, as well as to learning and memory (16). Aged rats exposed to EE show significant effects on hippocampal function. Enhanced hippocampal function results from activity-dependent increases in the levels of mGluR5, Homer1c, and phospho-p70S6 kinase (17).

Functional changes of neural circuitry are commonly addressed with long-term potentiation (LTP), which is related to memory formation and learning (18–20). EE improves hippocampal LTP with increased levels of BDNF (21). Exercise restores levels of neurotrophins and synaptic plasticity following spinal cord injury, which can play a role in recovery of locomotion following spinal cord injury (22). In Huntington's disease, EE rescues severe reductions in BDNF in the hippocampus and striatum, possibly by rescuing transcription or protein transport problems (23). In a rodent model of cerebral palsy, expression of synaptophysin increased in the primary motor cortex and ventral horn of the spinal cord after EE, as well as improving functional improvements (24).

The mechanisms underlying EE-mediated synaptic changes have yet to be fully elucidated in the striatum. Therefore, the aims of this study were to clarify the mechanisms of EE through the alteration of synaptic vesicle-associated proteins in the striatum, which provide a deeper understanding of the extent of functional improvement.

## Materials and methods

### Experimental procedures

#### Animals and enriched environment

**Figure 1:**
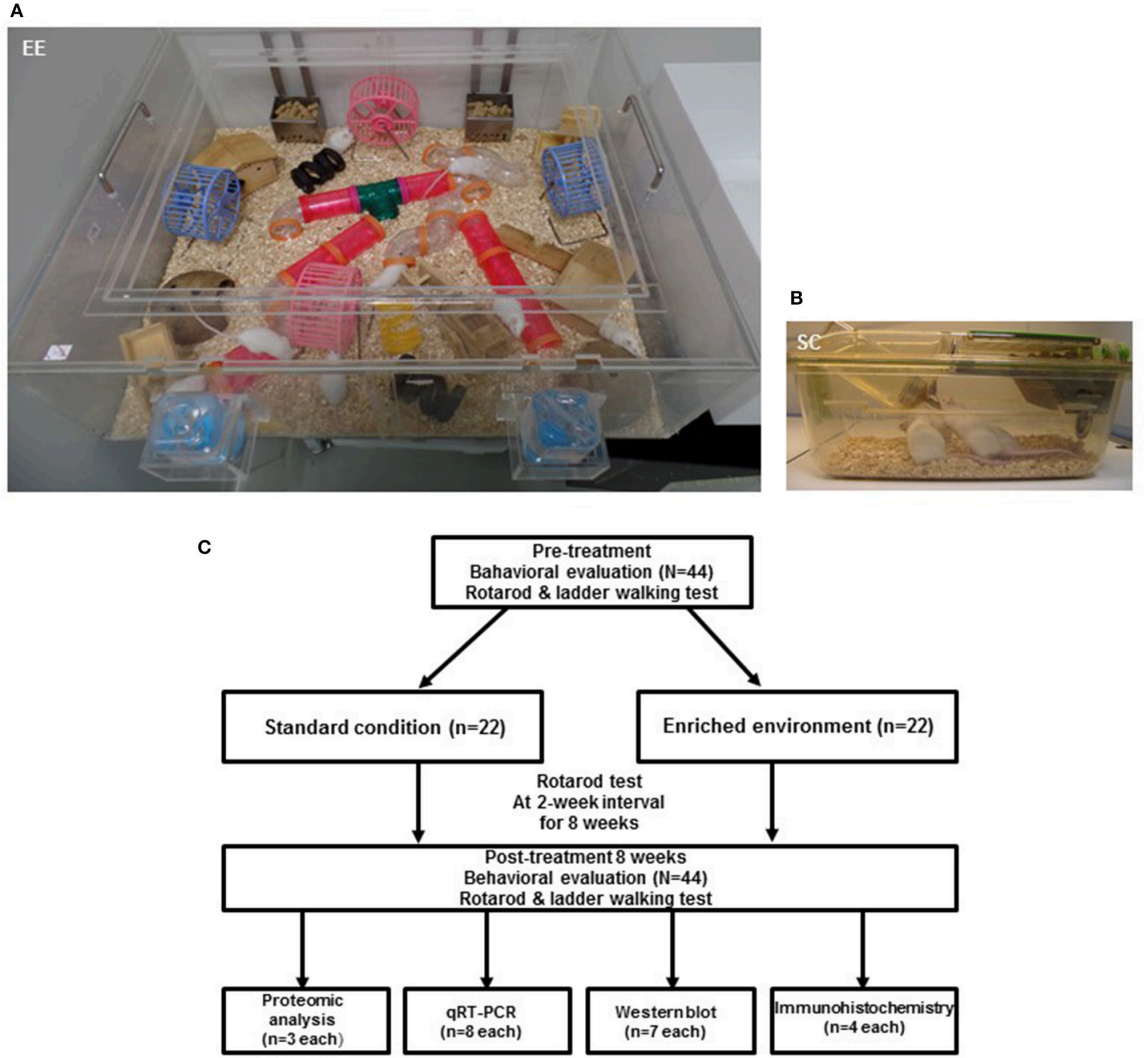
Experimental design for environmental enrichment. **(A)** The enriched environment (86 × 76 × 31 cm) including tunnels, shelters, toys, and running wheels for voluntary exercise and social interaction. **(B)** A standard cage (27 × 22.5 × 14 cm). **(C)** Experimental procedures and animal groups. A total of 44 mice were assigned to either standard cage (*n* = 22) or an enriched environment (*n* = 22) for 2 months. To investigate functional improvements in behavioral assessments, rotarod tests were performed at 2-week intervals, and ladder walking tests were performed post-intervention at 8 weeks. To identify the molecular mechanisms underlying the effect of EE, proteomic analysis (*n* = 3 per group) was carried out. To further validate the differentially expressed proteins, we performed qRT-PCR (*n* = 8 per group), western blot (*n* = 7 per group), and immunohistochemistry (*n* = 4 per group).

For two months, a total of 44 CD-1® (ICR) mice at 6 weeks of age were randomly housed to either standard condition [SC (*n* = 22, 7 male and 15 female) and an EE (*n* = 22, 10 male and 12 female)] until 14 weeks of age. The gender distributions between groups were not significant by Chi-square test (*p* = 0.353). EE mice freely accessed novel objects and large-scale social interaction (12~15 mice/cage) (Figure 1A) relative to controls (4 mice/cage) (Figure 1B). The time schedule and group assignment for this study is represented in Figure 1C. For all experiments, CD-1® (ICR) mice were housed in a facility accredited by the Association for Assessment and Accreditation of Laboratory Animal Care (AAALAC) and provided food and water ad libitum under alternating 12-h light/dark cycles, according to animal protection regulations.

#### Ethic statement

All procedures were reviewed and approved by the Animal Care and Use Committee of the Yonsei University College of Medicine (Permit number: 2017-0151). All procedures were in accordance with the guidelines of the National Institutes of Health's Guide for the Care and Use of Laboratory Animals. These regulations, notifications, and guidelines originated and were modified from the Animal Protection Law (2008), the Laboratory Animal Act (2008), and the Eighth Edition of the Guide for the Care and Use of Laboratory Animals (NRC 2011). They were sacrificed at 8 weeks after the housing conditions under ketamine (100 mg/kg) and xylazine (10 mg/kg) anesthesia by intraperitoneal injection. The brain tissues were harvested. All efforts were made to minimize animal suffering.

### Proteomic analysis

#### Protein sample preparation

After anesthetizing the animal, a 5–6 cm lateral incision through the integument and abdominal wall was conducted and then make a small incision to the diaphragm along the entire length of the rib cage. Avoiding potential damage to the lungs, A cut through the rib cage was performed to lift the sternum up. With 15-gauge blunt needle through the cut ventricle into the ascending aorta, an incision was then made to the animal's right atrium using iris scissors to create an outlet without damaging the descending aorta. Phosphate buffered saline (PBS) was used for perfusion of the heart. The head was disassembled using a pair of scissors. After removing the brain from the skull, the brain was washed with PBS for three times. Using a Leica M125 stereo microscope, striatum was carefully extracted for molecular studies. The striatum was snap frozen and stored in liquid nitrogen until use. Striatum in the region of mouse brain tissues were homogenized in 500 μl of cold RIPA buffer (0.25% sodium deoxycholate, 0.1% sodium dodecyl sulfate (SDS), 150 mM NaCl, 1% NP-40, 50 mM Tris-HCl (pH 7.4), 1 mM phenylmethylsulfonyl fluoride, 1 mM ethylenediaminetetraacetic acid) with a protease inhibitor cocktail (Sigma-Aldrich, St. Louis, MO, USA) on ice. The tissue lysates were centrifuged for 20 min at 13,000 g at 4°C. The supernatants containing the total brain protein extracts were collected at equal concentration and stored at 80°C until use. The protein content of the supernatant was determined using Bradford assay, according to the manufacturer's instructions.

#### In-gel digestion

Protein samples were separated with SDS-polyacrylamide gel electrophoresis (PAGE) on a 4–12% gradient Bis-Tris gel (Invitrogen, Carlsbad, CA, USA), and stained with GelCode Blue Stain Reagent (Pierce, Rockford, IL, USA). Each gel lane was cut into 10 pieces of equal size for tryptic digestion. The sliced gels were destained with 50% acetonitrile in 50 mM ammonium bicarbonate (Sigma), followed by dehydrating with 100% acetonitrile. A reduction step was performed with 5 mM dithiothreitol (Sigma) for 45 min at 60°C, followed by alkylation with 55 mM iodoacetamide (Sigma) for 30 min in darkness at room temperature. The resulting gels were digested by sequencing-grade modified trypsin (Promega, Madison, WI, USA) overnight at 37°C. Digested peptides were extracted from the gel with 5% formic acid in 50% acetonitrile. The extracted peptides were desalted using a C18 Spin-Column (Thermo Scientific, Foster City, CA, USA).

#### Chromatography-mass spectrometry analysis and data acquisition

Prepared samples were analyzed using Q Exactive mass spectrometer (Thermo Finnigan, San Jose, CA, USA) coupled with EASY-nLC 1000 (Thermo Finnigan). The tryptic peptides were loaded into a trap column (3 μm sized C18 resin into 75 μm sized C18 resin into 75 loaded into a trap column μm sized C18 resin into 75 μm sized C18 resin into 75 loaded il/min. The separated peptides were eluted by solvent A (0.1% formic acid in water) and solvent B (0.1% formic acid in acetonitrile) with a linear gradient for 60 min. Each mass spectrometry (MS) scan was followed by 8 MS/MS scans of the most abundant peak to the 8th-most abundant peak of the MS scan in the data-dependent method with dynamic exclusion options (repeat duration was 30 s and repeat count was set to 1). The normalized collision energy for MS/MS was 27%. To identify peptides, TurboSEQUEST (Thermo Electron, San Jose, CA, USA) was used to search MS/MS spectra against the UniProt database for forward sequences (17,098 Mus musculus protein sequence entries as of July 15, 2014). The mass tolerance was set to 10 ppm for precursor ions, 0.8 Da for fragment ions. Carbamidomethylation of cysteine (+57.0215 Da) and oxidation of methionine (+15.9949 Da) were set as the static modifications and variable modifications, respectively. Scaffold Q+S (version 4.3.4, Proteome Software Inc., OR, USA) was used to compute the probability of proteins and peptides 0.14. Search results were validated using the protein and peptide cut-off probabilities with greater than 99% and 95% probability, respectively. Spectral counts were calculated by Scaffold software after normalization by total spectral counts of each MS run for an estimate of expression fold change between the experimental groups (25).

#### Bioinformatics analysis

Gene ontology (GO) is widely used to describe protein function in a standardized format. GO analysis of the identified proteins was performed using the Database for Annotation, Visualization and Integrated Discovery (DAVID) 6.8 annotation tool to identify protein function. GO annotation protein group analysis according to their associated biological process, molecular function, and cellular components annotations. Biological process describes one or more organized assemblies for molecular function. To identify protein function, up-regulated protein-coding genes were used with biological process in DAVID annotation tool. Within several biological processes, proteins coding genes, involved in synaptic vesicle exocytosis, were focused for further validation studies.

### Molecular analysis

#### RNA sample preparation

Total RNA was prepared in the whole cell lysates using TRIzol reagent (Invitrogen Life Technologies, Carlsbad, CA, USA) according to the manufacturer's instructions. A nanodrop spectrophotometer (Thermo Fisher Scientific, Waltham, MA, USA) was used to confirm the quality and quantity of extracted RNA.

#### Quantitative real-time PCR (qRT-PCR)

Differentially expressed genes of interest related presynaptic scaffold proteins in the striatum were selected to validate by qRT-PCR. ReverTra Ace® qPCR RT Master Mix with gDNA Remover (Toyobo, Osaka, Japan) was used to synthesize cDNA with total RNA. Then, 2 μl of cDNA in a total volume of 20 μl was used in the following reaction. The qRT-PCR was performed in triplicate on a Light Cycler 480 (Roche Applied Science, Mannheim, Germany) using the Light Cycler 480 SYBR Green master mix (Roche), with thermocycler conditions as follows: amplifications were performed starting with a 300 s template preincubation step at 95°C, followed by 45 cycles at 95°C for 10 s, 53°C for 10 s, and 72°C for 10 s. The melting curve analysis began at 95°C for 5 s, followed by 1 min at 60°C. The specificity of the produced amplification product was confirmed by the examination of a melting curve analysis and showed a distinct single sharp peak with the expected Tm for all samples. A distinct single peak indicates that a single DNA sequence was amplified during qRT-PCR. We further validated the specificity of the primers for the product of interests with RT-PCR (Supplementary Figure 1). The primers were as follows: Rabphilin 3A-Forward, CCA AGA CAA CAG CAA CCT GC; Rabphilin 3A-Reverse, CAT TCC ACA CAG GGT TCC GA; Piccolo-Forward, TGG TTA CAG AGG GAT TGG TGG; Piccolo-Reverse, TGA CAG TTC AAG GCA GGG TC; SV2B-Forward, CTG TTC TGT GGG ACC AGC AT; SV2B-Reverse, AGA GAA GCA GCA GCC AGA AG; GAPDH was used as the internal control. Primers were designed using the NCBI primer blast with the parameters set to a product of 150–200 bp within the region surrounding the identified translocation. The expression of each gene of interest was obtained using the 2−ΔΔCt method. The expression level of each gene of interest was obtained using the 2−ΔΔCt method (26). Target-gene expression was normalized relative to the expression of GAPDH and represented as fold change relative to the control.

#### Western blot analysis

Western blot analysis was performed as previously described (13). To confirm the expression of SV2B, Rabphilin 3A, Piccolo, Synaptophysin and PSD-95 in the striatum in the EE and controls, 30 μg of total protein was extracted from the striatum of all mice and dissolved in sample buffer (60 mM Tris–HCl, pH 6.8, 14.4 mM b-mercaptoethanol, 25% glycerol, 2% SDS, and 0.1% bromophenol blue; Invitrogen), incubated for 10 min at 70°C, and separated on a 10% SDS reducing polyacrylamide gel (Invitrogen). Protein samples were separated with SDS-polyacrylamide gel electrophoresis (PAGE) on a 4–12% gradient Bis-Tris gel and Tris-Acetate gel (Invitrogen, Carlsbad, CA, USA). The separated proteins were further transferred onto a 0.45 μm invitrolonTM polyvinylidene difluoride (PVDF) filter paper sandwich using a XCell IITM Blot Module (invitrogen, Life Technologies, Carlsbad, CA, USA). The membranes were blocked for 1 h in Tris-buffered saline (TBS) (10 mM Tris-HCl, pH 7.5, 150 mM NaCl) plus 0.05% Tween 20 (TBST) containing 5% non-fat dry milk (Bio-Rad, Hercules, CA, USA) at room temperature, washed three times with TBST, and incubated at 4°C overnight with the following primary antibodies; anti-Rabphilin 3A (ab59259, Abcam) at a 1:1,000 dilution, anti-Piccolo (ab110427, Abcam) at a 1:1,000 dilution anti-SV2B (14624-1-AP, Proteintech) at a 1:1,000 dilution, anti-Synaptophysin(ab8049,Abcam) at a 1:1,000 dilution and anti-PSD95 (ab18258,Abcam) at a 1:1,000 dilution. After washing the blots three times with TBST, the blots were incubated for 1 h with horseradish peroxidase-conjugated secondary antibodies (1:5,000; Santa Cruz, CA, USA) at room temperature. The proteins were further washed three times with TBST and visualized with an enhanced chemiluminescence (ECL) detection system (Amersham Pharmacia Biotech, Little Chalfont, UK). Using ImageQuant™ LAS 4000 software (GE Healthcare Life Science, Chicago, IL, USA), western blot results were saved into TIFF image files, and then the images and the density of the band were analyzed and expressed as the ratio relative to the control band density using Multi-Gauge (Fuji Photo Film, version 3.0, Tokyo, Japan). To normalize the values of all samples to account for band intensity, the average band intensity for each mouse group was first calculated. The samples were normalized to the group average of controls. The value of the control group was set to 1 and was divided by the value of each individual mouse.

### Immunohistochemistry (IHC)

IHC was performed as previously described (16). The tissue was frozen in Surgipath FSC 22 clear frozen section compound (Leica Microsystems) using dry ice and isopentane. The harvested brain tissues were cryosectioned at 16-μm thickness along the coronal plane and immunohistochemistry staining was performed. At 8 weeks after EE, to confirm endogenous expression of piccolo, the brain sections of the striatum were immunostained Piccolo (1:400, ab110427, Abcam). Individual sections were also costained for the neuronal marker Tuj1 (1:400, Covance, Princeton, NJ, USA) or the astrocyte marker glial fibrillary acidic protein (GFAP; 1:400, Chemicon, Billerica, MA, USA) to confirm the identity of the specific neural lineage cells. The sections were incubated with Alexa Fluor® 488 goat anti-mouse (1:400, Invitrogen) and Alexa Fluor® 594 goat anti-rabbit (1:400, Invitrogen) secondary antibodies, then covered with Vectashield® mounting medium with 4C,6-diamidino-2-phenylindole (DAPI; Vector, Burlingame, CA, USA). The stained sections were analyzed using confocal microscopy (LSM700; Zeiss, Gottingen, Germany).

### Behavioral assessment

#### Rotarod performance

A rotarod was performed to assess motor coordination and balance. All animals received a pre-test performance at 5–6 weeks of age. Using constant speed at 48 rpm, rotarod tests were performed at 2-week intervals until the end of EE intervention. The latency to fall from the rod was measured twice during each test, and the maximum latency was limited to 300 s.

#### Ladder walking test

The ladder walking test can assess subtle disturbances of motor function through qualitative and quantitative analysis of walking (27). This test was performed at 5–6 weeks of age as a baseline study. The ladder walking test was performed 8 weeks after intervention. In the ladder walking test, the mice were required to walk a distance of 1 m four times on a horizontal ladder with metal rungs (Jeung Do Bio & Plant Co., Seoul, South Korea) located at differing distances apart. The number of slips in each forelimb was measured using videotape analysis (28). The variance between the control and EE groups was calculated as the difference in the percentage of slips on the transverse rungs of the ladder relative to the total number of steps taken by each forelimb of the EE mice compared to the controls.

### Statistical analysis

Statistical analyses were performed using Statistical Package for Social Sciences (SPSS) software (IBM Corporation, Armonk, NY, USA; version 23.0). Data are expressed as the mean ± standard error of the mean (SEM). At baseline, the independent *t*-test and Chi-square test were used for comparison between control and EE group. An independent *t*-test was used for the comparison of continuous variables between 2 groups in qRT-PCR and ladder walking test. A *p*-value < 0.05 was considered statistically significant. A two-way repeated measure analysis of variance (ANOVA) test was used to examine the main and interaction effects within and between groups (5 × 2 factorial design) for the rotarod test. When the time effect occurred, *post hoc* analysis was used to find where the significant differences were identified at *p*-value of < 0.01 using Bonferroni adjustment as multiple pairwise comparison.

## Results

### Environmental enrichment improves locomotor performance

**Figure 2:**
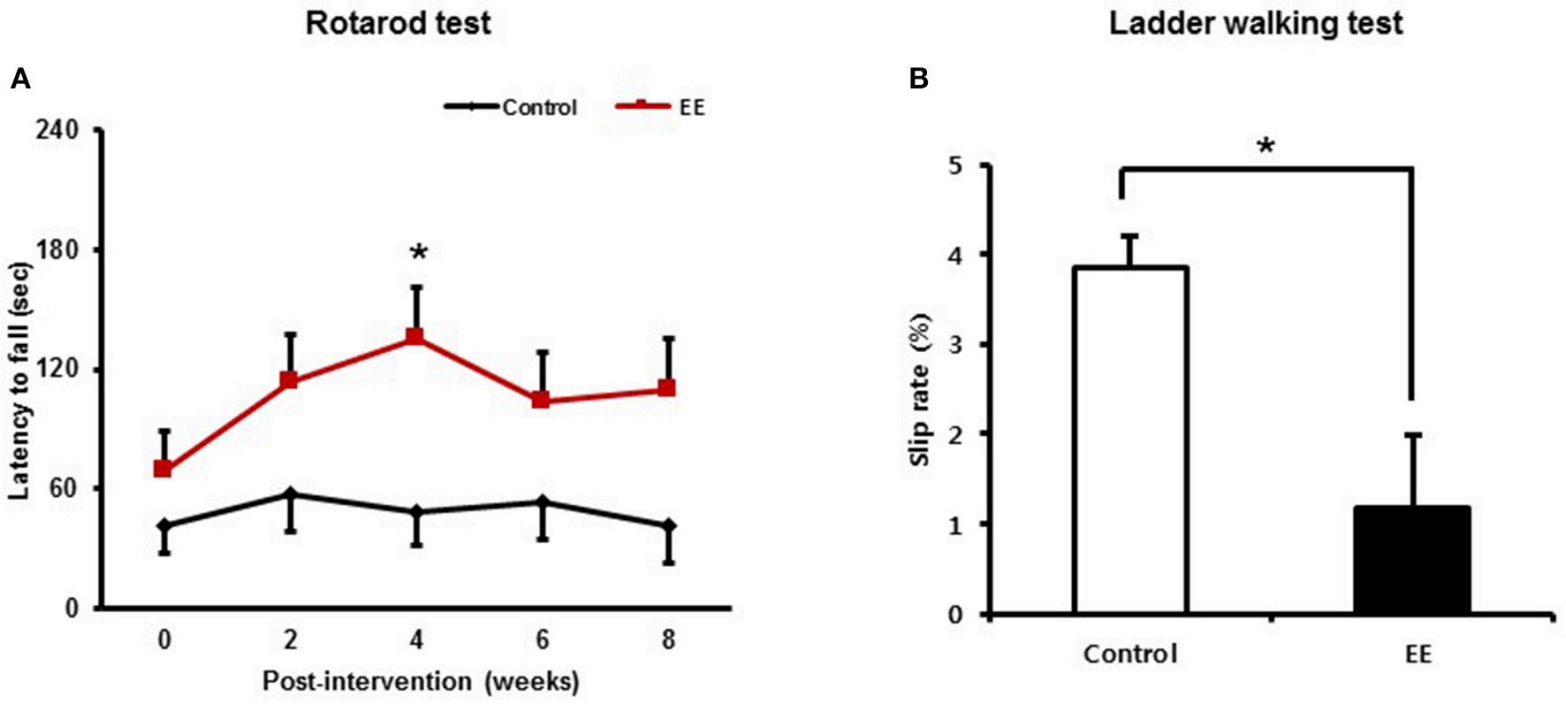
Environmental enrichment improves locomotor performance. **(A)** In the rotarod test, locomotive performance was enhanced at 4 weeks after exposure to EE compared to baseline (*p < 0.01 by Bonferroni-corrected pairwise comparison after two-way repeated measures ANOVA). **(B)** In the ladder walking test, EE mice showed lower percentage of total slips among total steps with both forelimbs at 8 weeks post-intervention compared to the controls (*p < 0.05 by independent *t*-test). Values are presented as mean ± S.E.M.

After mice were exposed to either EE or control for 2 months, rotarod tests were performed every 2 weeks to determine if EE improved motor function at a constant speed of 48 rpm. At baseline, there were no significant difference in the results of rotarod test between the EE group (69.28 ± 19.52 s) and control (41.40 ± 13.71 s; *p* = 0.249) A significant time x group interaction of the rotarod test was not revealed [Wilk's Lambda = 0.811, *F*_(4, 39.00)_ = 2.275, *p* = 0.079]. For the main effect of time [Wilk's Lambda = 0.654, *F*_(4, 39.00)_ = 5.149, *p* = 0.002] and group effect [*F*_(1, 42.00)_ = 5.176, *p* = 0.028] were revealed by a two-way repeated measure ANOVA analysis. Within groups, the rotarod performance was increased after 4 weeks intervention (135.30 ± 26.23 s) compared to baseline (69.28 ± 19.52 s; *p* = 0.002) in EE group, while this locomotive activity was not improved over the time in control group by multiple pairwise comparison (Figure 2A).

The ladder walking tests were carried out at baseline and at 8 weeks after intervention to evaluate whether EE improves fine motor function on the ladder. EE mice exhibited lower forelimb slip rate (1.18 ± 0.39%) at 8 weeks post-intervention, compared to controls (4.13 ± 0.62%; *p* < 0.01) by independent *t*-test (Figure 2B). These results identified from the rotarod test and the ladder walking test suggest that EE can improve locomotor function and fine motor function.

### Bioinformatic analysis of differentially expressed proteins (DEPs)

We conducted proteomic analysis to identify DEPs in mice exposed to EE compared to controls. We identified 520 up-regulated proteins and 680 down-regulated proteins. Among these proteins, 54 up-regulated proteins were 1.5-fold higher and 62 down-regulated proteins were 1.5-fold lower in mice exposed to EE (Table 1 and Supplementary Table 1). Gene ontology analyses of biological processes with up-regulated proteins in the striatum were performed using DAVID programs (Table 2). Biological process analysis indicated that up-regulated proteins in the striatum of mice exposed to EE were related to “synaptic vesicle exocytosis.” The GO terms for this biological process commonly included Synaptic vesicle glycoprotein 2B (SV2B), Rabphilin-3A, and Piccolo.

**Table 1:** Up-regulated proteins by exposure to EE in striatum.

**Accession**	**Description**	**Gene symbol**	**Fold change**
E9Q3W4	Plectin	PLEC	3.45
Q8K0U4	Heat shock 70 kDa protein 12A	HSPA12A	2.91
Q8K212	Phosphofurin acidic cluster sorting protein 1	PACS1	2.73
Q9Z2I0	LETM1 and EF-hand domain-containing protein 1, mitochondrial	LETM1	2.56
Q91V89	Protein Ppp2r5d	PPP2R5D	2.36
Q3UGX2	Protein Spnb1	SPNB1	2.26
P84084	ADP-ribosylation factor 5	ARF5	2.17
Q99PU5	Long-chain-fatty-acid–CoA ligase ACSBG1	ACSBG1	2.09
Q9R1V6-19	Isoform 18 of Disintegrin and metalloproteinase domain-containing protein 22	ADAM22	2.09
Q9CZW5	Mitochondrial import receptor subunit TOM70	TOMM70A	2.07
Q8BG39	Synaptic vesicle glycoprotein 2B	SV2B	2.03
Q9ESN6	Tripartite motif-containing protein 2	TRIM2	1.96
P61205	ADP-ribosylation factor 3	ARF3	1.93
Q3UNH4	G protein-regulated inducer of neurite outgrowth 1	GPRIN1	1.93
P68404-2	Isoform Beta-II of Protein kinase C beta type	PRKCB	1.88
Q64521	Glycerol-3-phosphate dehydrogenase, mitochondrial	GPD2	1.87
P29341	Polyadenylate-binding protein 1	PABPC1	1.85
Q64514-2	Isoform Short of Tripeptidyl-peptidase 2	TPP2	1.83
Q8BKC5	Importin-5	IPO5	1.81
Q8BH59	Calcium-binding mitochondrial carrier protein Aralar1	SLC25A12	1.76
P35564	Calnexin	CANX	1.76
Q61941	NAD(P) transhydrogenase, mitochondrial	NNT	1.76
Q3UHL1	CaM kinase-like vesicle-associated protein	CAMKV	1.73
P47708	Rabphilin-3A	RPH3A	1.72
A2AN08-3	Isoform 3 of E3 ubiquitin-protein ligase UBR4	UBR4	1.71
Q61656	Probable ATP-dependent RNA helicase DDX5	DDX5	1.71
Q7M6W1	RTN1-C	RTN1	1.71
B8QI35	Liprin-alpha 3	PPFIA3	1.70
B0V2N1-4	Isoform 4 of Receptor-type tyrosine-protein phosphatase S	PTPRS	1.70
E9Q2A6	Protein-tyrosine kinase 2-beta	PTK2B	1.70
Q8BP47	Asparagine–tRNA ligase, cytoplasmic	NARS	1.70
P50516	V-type proton ATPase catalytic subunit A	ATP6V1A	1.69
P24527	Leukotriene A-4 hydrolase	LTA4H	1.68
Q61290	Voltage-dependent R-type calcium channel subunit alpha-1E	CACNA1E	1.68
P11881-8	Isoform 8 of Inositol 1,4,5-trisphosphate receptor type 1	ITPR1	1.62
Q8R366	Immunoglobulin superfamily member 8	IGSF8	1.61
E9QN14	SLIT-ROBO Rho GTPase-activating protein 3	SRGAP3	1.59
Q9D0E1-2	Isoform 2 of Heterogeneous nuclear ribonucleoprotein M	HNRNPM	1.59
P40142	Transketolase	TKT	1.59
Q8BX70-2	Isoform 2 of Vacuolar protein sorting-associated protein 13C	VPS13C	1.58
Q8CIE6	Coatomer subunit alpha	COPA	1.56
Q8BMF3	NADP-dependent malic enzyme, mitochondrial	ME3	1.56
Q6PB66	Leucine-rich PPR motif-containing protein, mitochondrial	LRPPRC	1.55
D3Z7P3	Glutaminase kidney isoform, mitochondrial	GLS	1.53
Q9QYX7-2	Isoform 2 of Protein piccolo	PCLO	1.53
Q60597-2	Isoform 2 of 2-oxoglutarate dehydrogenase, mitochondrial	OGDH	1.51
Q4FE56	Ubiquitin carboxyl-terminal hydrolase	USP9X	1.51
O70161-3	Isoform 3 of Phosphatidylinositol 4-phosphate 5-kinase type-1 gamma	PIP5K1C	1.51
P32037	Solute carrier family 2, facilitated glucose transporter member 3	SLC2A3	1.51
Q91ZA3	Propionyl-CoA carboxylase alpha chain, mitochondrial	PCCA	1.51
E9Q035	Protein Gm20425	GM20425	1.51
Q7TMY8-4	Isoform 4 of E3 ubiquitin-protein ligase HUWE1	HUWE1	1.51
D3YU23	Latrophilin-3	LPHN3	1.51
Q8C1A5	Thimet oligopeptidase	THOP1	1.51

*EE, Environmental Enrichment*.

**Table 2:** Gene ontology for biological process by exposure to EE in striatum.

**Term**	**Count**	**%**	***P*-Value**	**Genes**
GO:0099003~vesicle mediated transport in synapse	4	0.047	0.002	**SV2B**, **RPH3A**, **PCLO**,CANX
GO:0051641~cellular localization	15	0.177	0.002	**SV2B**, **RPH3A**, **PCLO**, PACS1, PPFIA3, COPA, PIP5K1C, CANX, ITPR1, HNRNPM, HUWE1, VPS13C, PTK2B, IPO5, CACNA1E
GO:0006890~retrograde vesicle-mediated transport, Golgi to ER	3	0.035	0.003	COPA, ARF3, ARF5
**GO:0099504**~**synaptic vesicle cycle**	**4**	**0.047**	**0.003**	**SV2B, RPH3A, PCLO**
GO:0099643~signal release from synapse	4	0.047	0.003	**SV2B**, **RPH3A**, **PCLO**, PPFIA3
GO:0007269~neurotransmitter secretion	4	0.047	0.003	**SV2B**, **RPH3A**, **PCLO**,PPFIA3
GO:0097480~establishment of synaptic vesicle localization	4	0.047	0.003	**SV2B**, **RPH3A**, **PCLO**,CANX
**GO:0048489**~**synaptic vesicle transport**	**4**	**0.047**	**0.003**	**SV2B, RPH3A, PCLO**
GO:0099531~presynaptic process involved in chemical synaptic transmission	4	0.047	0.004	**SV2B**, **RPH3A**, **PCLO**, PPFIA3
**GO:0097479**~**synaptic vesicle localization**	**4**	**0.047**	**0.004**	**SV2B, RPH3A, PCLO**
GO:0051649~establishment of localization in cell	12	0.142	0.004	**SV2B**, **RPH3A**, **PCLO**, PACS1, COPA, PPFIA3, HUWE1, VPS13C, PTK2B, IPO5, CANX, ITPR1
GO:0015031~protein transport	12	0.142	0.004	**SV2B**, **RPH3A**, **PCLO**, PACS1, COPA, HNRNPM, HUWE1, VPS13C, ARF3, IPO5, CACNA1E, ARF5
GO:0019362~pyridine nucleotide metabolic process	4	0.047	0.005	GPD2, NNT, TKT, OGDH
GO:0046496~nicotinamide nucleotide metabolic process	4	0.047	0.005	GPD2, NNT, TKT, OGDH
GO:0072524~pyridine-containing compound metabolic process	4	0.047	0.006	GPD2, NNT, TKT, OGDH
GO:0006733~oxidoreduction coenzyme metabolic process	4	0.047	0.007	GPD2, NNT, TKT, OGDH
GO:0045184~establishment of protein localization	12	0.142	0.009	**SV2B**, **RPH3A**, **PCLO**, PACS1, COPA, HNRNPM, HUWE1, VPS13C, ARF3, IPO5, CACNA1E, ARF5
GO:0016192~vesicle-mediated transport	9	0.106	0.010	**SV2B**, **RPH3A**, **PCLO**, PACS1, COPA, ARF3, PIP5K1C, ARF5, CANX
GO:0006836~neurotransmitter transport	4	0.047	0.011	**SV2B**, **RPH3A**, **PCLO**, PPFIA3
GO:0051650~establishment of vesicle localization	4	0.047	0.011	**SV2B**, **RPH3A**, **PCLO**, CANX
GO:0001505~regulation of neurotransmitter levels	4	0.047	0.012	**SV2B**, **RPH3A**, **PCLO**, PPFIA3
GO:0051648~vesicle localization	4	0.047	0.013	**SV2B**, **RPH3A**, **PCLO**, CANX
GO:0048193~Golgi vesicle transport	4	0.047	0.016	PACS1, COPA, ARF3, ARF5
**GO:0016079**~**synaptic vesicle exocytosis**	**3**	**0.035**	**0.016**	**SV2B, RPH3A, PCLO**
GO:0099537~trans-synaptic signaling	6	0.071	0.016	**SV2B**, **RPH3A**, **PCLO**, PPFIA3, PTK2B, GLS
GO:0007268~chemical synaptic transmission	6	0.071	0.016	**SV2B**, **RPH3A**, **PCLO**, PPFIA3, PTK2B, GLS
GO:0098916~anterograde trans-synaptic signaling	6	0.071	0.016	**SV2B**, **RPH3A**, **PCLO**, PPFIA3, PTK2B, GLS
GO:0099536~synaptic signaling	6	0.071	0.016	**SV2B**, **RPH3A**, **PCLO**, PPFIA3, PTK2B, GLS
GO:0008104~protein localization	13	0.153	0.017	**SV2B**, **RPH3A**, **PCLO**, PACS1, COPA, HNRNPM, HUWE1, VPS13C, ARF3, IPO5, PIP5K1C, CACNA1E, ARF5
GO:1902369~negative regulation of RNA catabolic process	2	0.024	0.018	PABPC1, LRPPRC
GO:0033036~macromolecule localization	14	0.165	0.019	**SV2B**, **RPH3A**, **PCLO**, PACS1, COPA, HNRNPM, HUWE1, VPS13C, ARF3, IPO5, PIP5K1C, CACNA1E, ARF5, LRPPRC
GO:0009117~nucleotide metabolic process	6	0.071	0.019	GPD2, ATP6V1A, NNT, PTK2B, TKT, OGDH
GO:0006753~nucleoside phosphate metabolic process	6	0.071	0.021	GPD2, ATP6V1A, NNT, PTK2B, TKT, OGDH
GO:0046903~secretion	8	0.094	0.021	**SV2B**, **RPH3A**, **PCLO**, COPA, PPFIA3, PIP5K1C, CACNA1E, PRKCB
GO:0021766~hippocampus development	3	0.035	0.021	USP9X, PTPRS, OGDH
GO:0043648~dicarboxylic acid metabolic process	3	0.035	0.021	ME3, GLS, OGDH
GO:0044248~cellular catabolic process	9	0.106	0.027	GPD2, HUWE1, PTK2B, USP9X, GLS, UBR4, LTA4H, PABPC1, LRPPRC
GO:0055086~nucleobase-containing small molecule metabolic process	6	0.071	0.028	GPD2, ATP6V1A, NNT, PTK2B, TKT, OGDH
GO:0023061~signal release	5	0.059	0.029	**SV2B**, **RPH3A**, **PCLO**, PPFIA3, CACNA1E
**GO:0017156**~**calcium ion regulated exocytosis**	3	0.035	0.029	**SV2B, RPH3A, PCLO**
GO:0032940~secretion by cell	7	0.083	0.034	**SV2B**, **RPH3A**, **PCLO**, PPFIA3, PIP5K1C, CACNA1E, PRKCB
GO:0021549~cerebellum development	3	0.035	0.035	USP9X, PTPRS, OGDH
GO:0021761~limbic system development	3	0.035	0.037	USP9X, PTPRS, OGDH
GO:0019637~organophosphate metabolic process	7	0.083	0.038	GPD2, ATP6V1A, NNT, PTK2B, PIP5K1C, TKT, OGDH
GO:0022037~metencephalon development	3	0.035	0.042	USP9X, PTPRS, OGDH
GO:0051235~maintenance of location	4	0.047	0.043	VPS13C, PTK2B, PIP5K1C, ITPR1
GO:0055085~transmembrane transport	7	0.083	0.046	SLC25A12, ATP6V1A, PTK2B, SLC2A3, CACNA1E, **SV2B**, ITPR1
GO:0006887~exocytosis	4	0.047	0.046	**SV2B**, **RPH3A**, **PCLO**, PIP5K1C
GO:0006732~coenzyme metabolic process	4	0.047	0.047	GPD2, NNT, TKT, OGDH

*EE, Environmental Enrichment; SV2B, Synaptic Vesicle Glycoprotein 2B; RPH3A, Rabphilin-3A; PCLO, Piccolo. Bold characters and values are associated with the interested genes, SV2B, RPH3A, and PCLO*.

### Validation of DEPs

**Figure 3:**
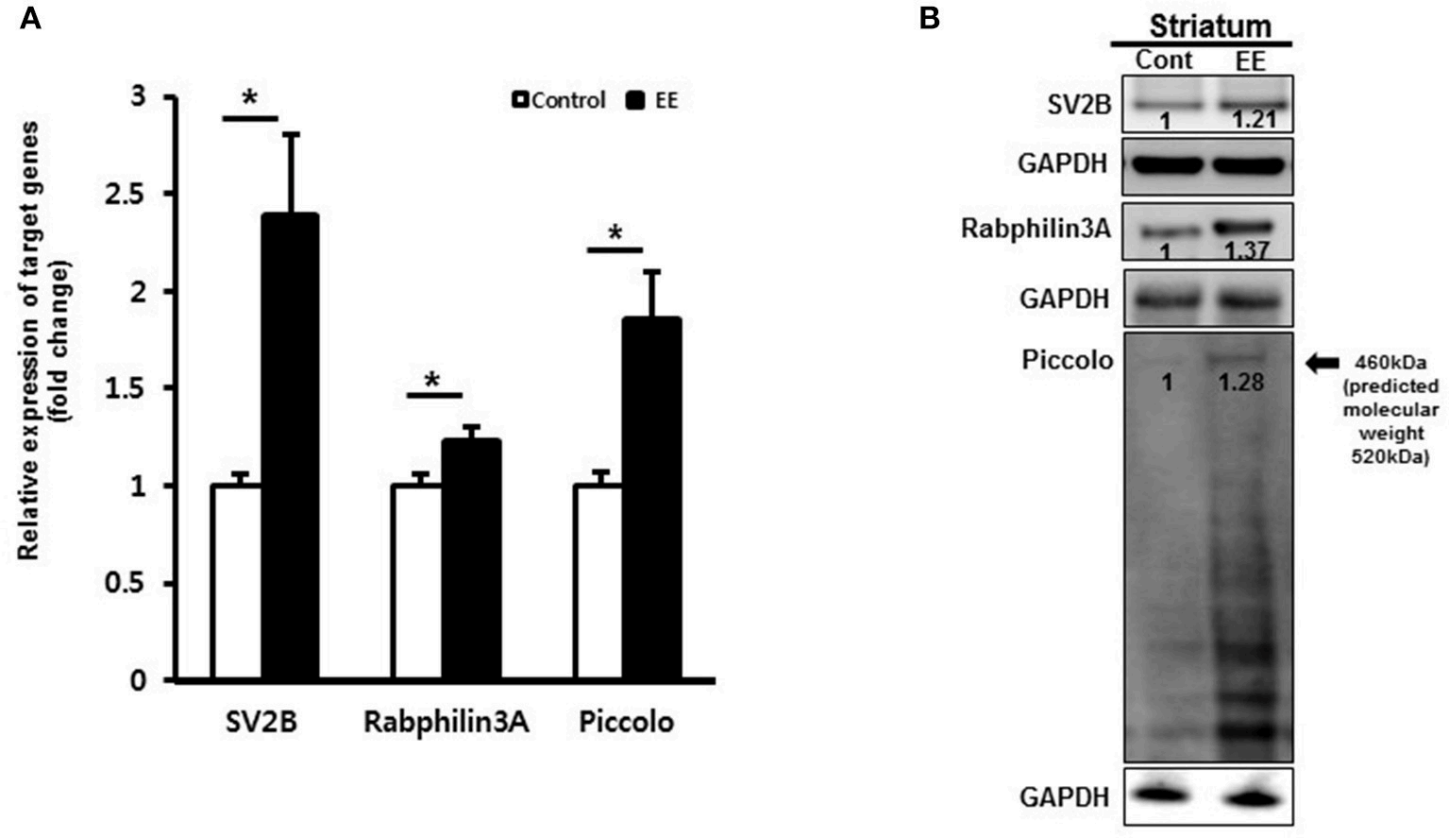
Validation of synaptic vesicle-associated proteins. **(A)** Validation of DEPs by qRT-PCR. The mRNA levels of SV2B, Rabphilin-3A and Piccolo were significantly upregulated in the EE mice compared to controls (*p* < 0.05). **(B)** Validation of DEPs by western blotting in striatum. The protein expression of SV2B, Rabphilin-3A and Piccolo showed upregulation at 8 weeks after exposure to EE.

We examined mRNA expression with qRT-PCR in EE mice compared to controls. qRT-PCR analysis of striatal tissue confirmed that the mRNAs of SV2B, Rabphilin-3A and Piccolo were significantly upregulated in EE mice relative to controls (Figure 3A). To validate the DEPs, total protein was extracted from the striatum of mouse brains and further examined using western blotting. EE mice showed upregulated expression of SV2B, Rabphilin-3A and Piccolo in the striatum compared to the control group (Figure 3B).

### Characterization of altered protein expression as a result of EE

**Figure 4:**
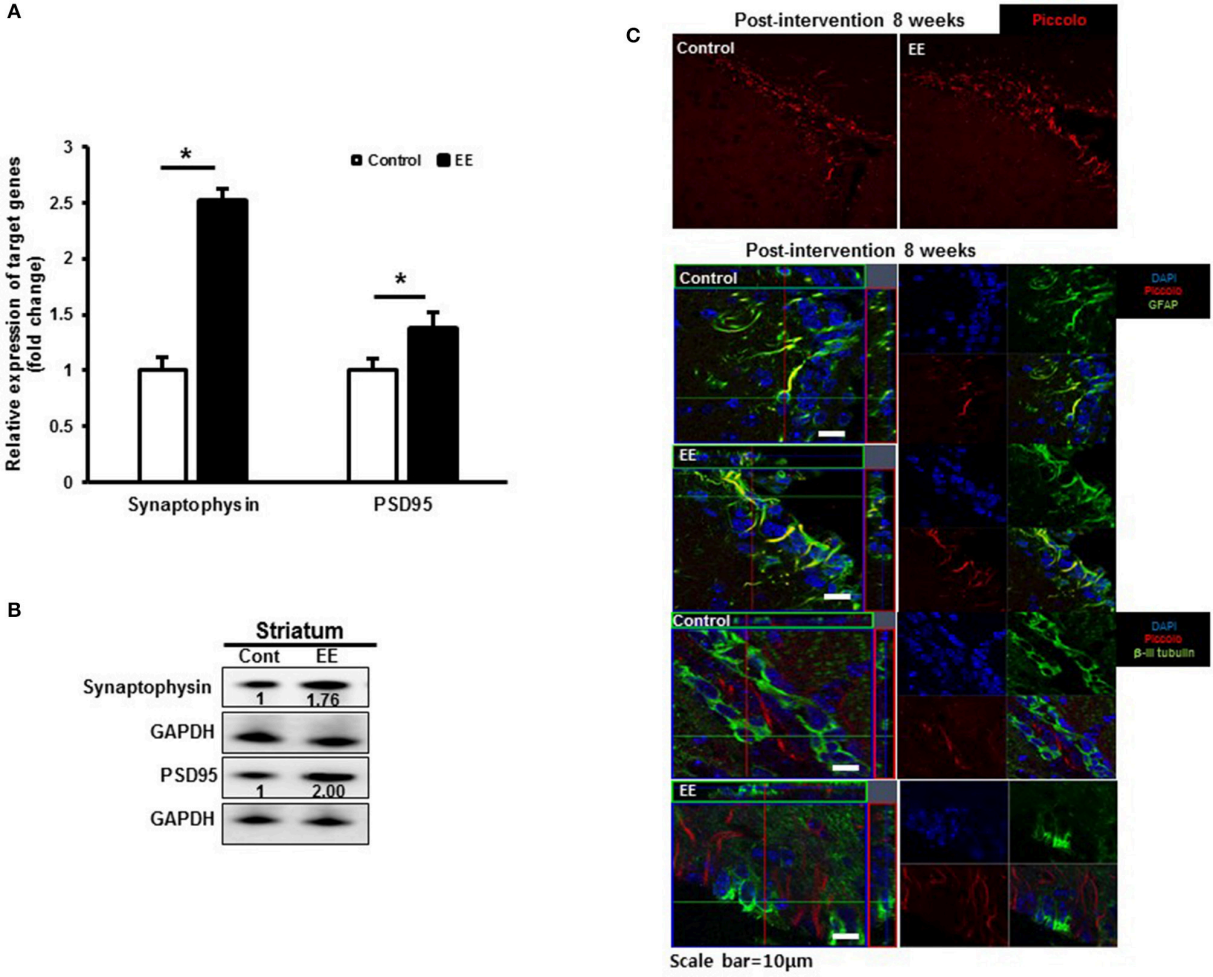
Validation of synaptic density proteins and the Piccolo expression. **(A)** Validation of synaptic density protein by qRT-PCR. The significant upregulation of synaptophysin and PSD95 was observed in EE mice compared to controls at RNA level. **(B)** Validation of synaptic density proteins by western blot. The upregulation of synaptophysin and PSD95 was observed in EE mice compared to controls at protein level. **(C)** Histological assessment for Piccolo. EE enhanced the expression of Piccolo in the subventricular zone. Fractions of Piccolo^+^ cells show astroglial phenotypes such as GFAP^+^ after long-term exposure to EE. However, the fraction of Piccolo was not co-labeled with β-III tubulin.

Many of the identified proteins showed significantly different expression levels between the control group and EE group. We focused on determining how EE exposure promotes synaptic plasticity using the results of GO analysis. The GO terms for the biological processes commonly included SV2B, Rabphilin-3A and Piccolo. The RNA expression of synaptophysin, a presynaptic marker and PSD95, a postsynaptic marker was significantly upregulated in EE mice compared to controls (Figure 4A). Moreover, the protein expression of synaptophysin and PSD95 was upregulated in EE mice compared to controls (Figure 4B). The effect of treatment with EE in inducing endogenous Piccolo expression in the striatum was assessed immunohistologically by quantifying the density of Piccolo^+^ cells at 8 weeks after EE. At 8 weeks after EE, the density of Piccolo in the striatum was increased in EE mice compared with controls (Figure 4C). In addition, a fraction of Piccolo had the astroglial phenotype as confirmed by co-labeling with GFAP^+^ cells, but not with βIII tubulin^+^ cells, suggesting that Piccolo expression is enhanced by astroglial lineage cells with long-term exposure to EE (Figure 4C).

## Discussion

We used proteomics analysis along with KEGG-pathway and GO analyses to determine how long-term exposure to EE promotes synaptic plasticity in this study. Here, we examined the effects of EE on protein expression and alterations in signaling pathways in the striatum where is important for functions such as motor control and coordination (28, 29). In addition, we conducted qRT-PCR and western blot analyses to validate the DEPs. Among the upregulated proteins, SV2B, Rabphilin-3A and Piccolo have been identified as cytomatrix proteins that are specifically localized at the active zone (30). These proteins are likely to function as scaffolding proteins of the active zone that participate in synaptic vesicle-recycling and exocytosis machinery. The upregulation of these proteins is also closely related to behavioral improvements, as shown in rotarod and ladder walking tests in this study. Previous studies suggested that exercise induces alterations in the expression of synaptic proteins in the motor cortex and striatum, which is responsible for the learning of complex motor tasks (31), and synaptic scaffolding proteins in the striatum are implicated in improving motor performance through activating synaptic glutamate receptors and calcium influx (32).

Among the proteins upregulated in the brains of mice exposed to EE that were common in the identified GO terms, Rabphilin-3A, an evolutionarily conserved synaptic vesicle-associated protein, attaches to synaptic vesicle membranes via its N-terminus (33). Previous studies have shown that Rabphilin-3A has a special interaction with Ca^2+^ and synaptotagmin (33–35) and binds to other synaptic vesicle proteins such as Rab3A and Rab3C in a GTP-dependent fashion (35, 36). Additionally, Rabphilin-3A interacts with PIP2/Ca^2+^ and SNAP25 via its C2B domain and regulates synaptic activity by interacting with other presynaptic active zone molecules via a calcium-related pathway (37, 38). Previoust studies showed that SV2B is associated with calcium-regulated exocytosis and synaptic activity and regulates the interactions of synaptotagmin 1 and t-SNARE proteins (39–41). These accumulating evidences suggest that Rabphilin-3A and SV2B regulate synaptic vesicle activity in a calcium-dependent manner and therefore improve locomotor improvement through interacting with other presynaptic proteins (35, 36, 42).

Another GO term-related protein, Piccolo, which is one of the cytomatrix proteins at the presynaptic active zone. Piccolo is mainly deposited at neurotransmitter release sites, suggesting that it may participate in presynaptic plasticity by regulating synaptic vesicle cycles (33). Many previous studies revealed that the Zn^2+^ fingers of Piccolo interact with other proteins such as prenylated Rab acceptor protein PRA1 and synaptobrevin 2/VAMP2, which are involved in synaptic vesicle formation and exocytosis (43–45). Piccolo is a scaffold protein that constitutes the presynaptic active zone and interacts with several proteins of other presynaptic active zones such as Munc13, RIM, Liprin-a, ELKS/CAST, RBP, and Bassoon to form the core active zone complex (46). Other presynaptic active zone proteins that interact with Piccolo play roles in maintaining the structural function of the presynaptic active zone, regulating exocytosis and endocytosis. Therefore, upregulation of Piccolo activates the presynaptic active zone by regulating exocytosis and endocytosis, which indicates that it regulates synaptic plasticity. Moreover, the study conducted by Ibi et al, suggested that Piccolo knockdown-induced mice show an impairment in spatial learning, and Piccolo has an important role in synaptic plasticity in the CA1 region of hippocampus (47). Taken together, these findings indicate that Piccolo is a scaffolding protein of the active zone that may engage in constructing large protein interaction networks for the synaptic vesicle cycle. The upregulation of Piccolo may be related to locomotor improvement through tightly regulating exocytosis and endocytosis of presynaptic vesicles and creating large connections with other presynaptic proteins (41–44, 48).

A fraction of Piccolo was expressed in GFAP^+^ cells, suggesting that astroglial-lineage cells were enhanced with the Piccolo expression after long-term exposure to EE. The upregulated expression of Piccolo and colocalization between astrocytes and Piccolo may be responsible for synaptic plasticity via astrocyte modification. Previous studies have shown astrocytes have been implicated in the forms of plasticity such as long-term potentiation, and its modification depends on neuronal activity (49–51). For instance, astrocytes sense neuronal activity by elevating their intracellular calcium in response to neurotransmitters and may communicate with neurons (49). Astrocytes, the most abundant cell type in the animal brain, play a critical role in the modulation of synaptic transmission by forming the tripartite synapses in between neurons (52, 53). Growing evidence suggests that this communication between astrocytes and neurons plays a role in synaptic plasticity and neuroprotection (53–55). Previous studies highlighted the effects of EE on astrocytic modification in terms of gliogenesis, morphological change, and antigen expression in young rats as well as aged rats, showing that the increased number of astrocytes and the alteration of astrocyte morphology were noted in the dorsal hippocampus after long-term exposure to EE (56–59). EE mediates not only astrocytic modification, but also synaptic plasticity-associated activities such as cortical thickness (14), dendritic branching (60), spine density (14, 60), LTP (61), and neurogenesis (62). Therefore, EE-mediated enhancement of Piccolo expression can affect astrocytes as well as neurons, facilitating synaptic transmission and neuronal plasticity. Identification of the molecular cascade that bridges astrocytic calcium signaling, Connexin-30, a gap junction protein, could be a major mechanism for astrocytic involvement of experience-dependent plasticity (63). Connexin-30 is elevated after EE (15) and positively regulated by neural activity (64). Previous studies have also found that physical and social components of EE treatment enhanced astrocyte proliferation and increased the expression of BDNF, which might contribute to improved neurological outcomes in stroke animals, and physical activity plays a more important role in EE treatment after cerebral ischemia/reperfusion injury (65, 66).

Taken together, the upregulation of presynaptic vesicle-associated proteins such as Rabphilin-3A, Piccolo, and SV2B may be related to the fine-tuning control of vesicle fusion events, enabling more precise movement and locomotor performance improvement. The evidence suggests that EE enhances neurobehavioral functions, and that these effects might be mediated by the upregulation of presynaptic scaffolding proteins in the striatum. Although molecular analyses have not yet revealed any neurophysiological linkage, our results suggest a possible mechanism of functional recovery of EE that is associated with upregulation of synaptic proteins in the striatum (67).

As a limitation of this study, our data are restricted to the striatal region. Since the striatum contains many crossing axonal packages, it may be difficult to determine the exact location of astrocyte modification. Further studies should be conducted whether the colocalization between GFAP and synaptic vesicle-associated proteins is also observed in other brain regions.

## Conclusion

**Figure 5:**
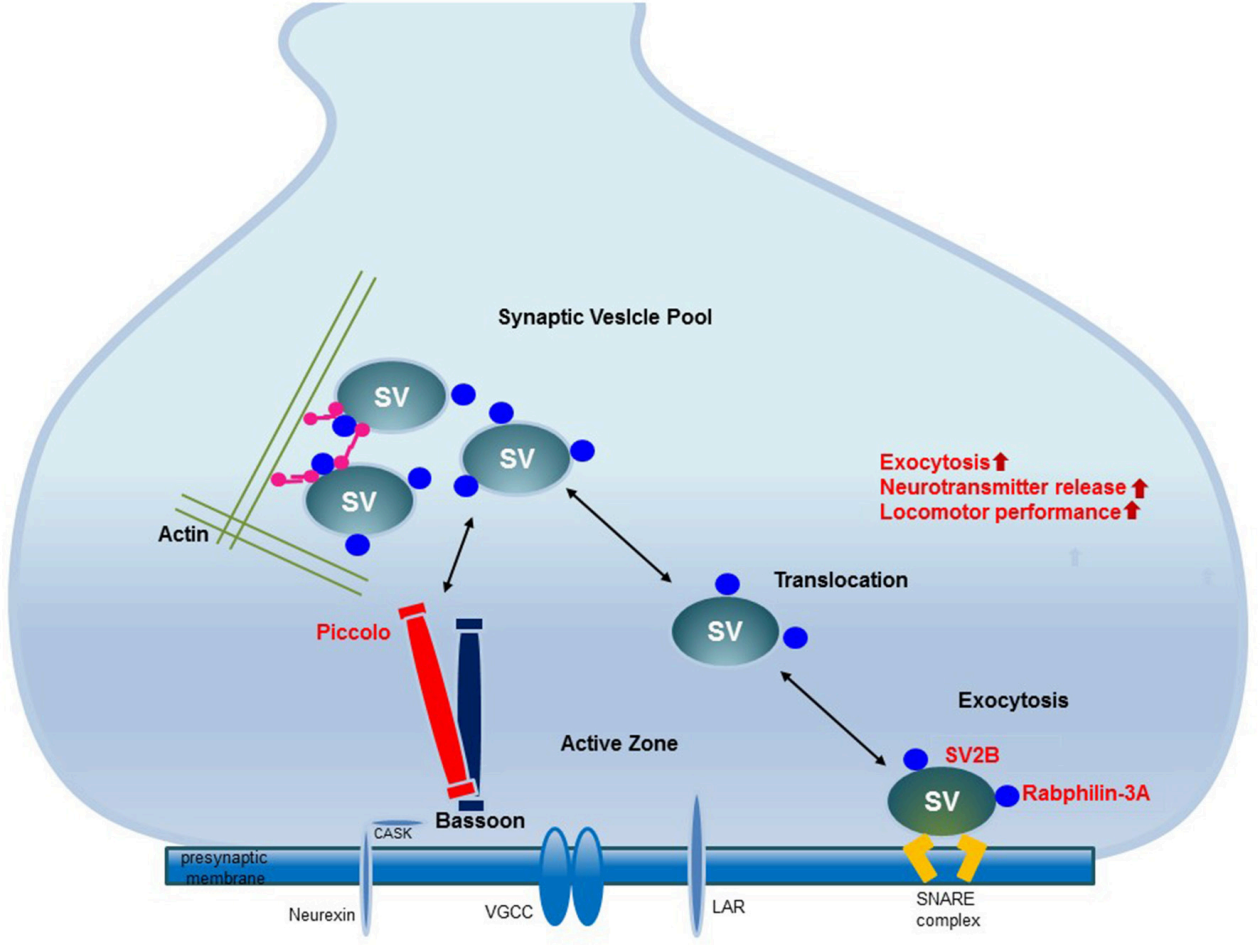
The mechanism underlying synaptic plasticity induced by environmental enrichment. The increased expression of synaptic vesicle-associated proteins leads to neural plasticity due to the activation of synaptic proteins and synaptic vesicle fusion. Among the presynaptic active zone proteins, Piccolo interacts with other presynaptic active zone proteins to organize a super-molecular complex. SV2B is involved in exocytosis. Rabphilin-3A regulates exocytosis and endocytosis in the presynaptic zone. The increased expression of these proteins promotes their interactions with other proteins in the presynaptic active zone, may regulate synaptic vesicle activity and induce synaptic plasticity. These upregulated presynaptic proteins may be responsible for improved locomotor performance.

EE upregulates the proteins related to synaptic vesicle transport and exocytosis in the striatum. The upregulation of these proteins may be related to the fine-tuning control of vesicle fusion events and therefore improve locomotor performance. These upregulated proteins interact with other presynaptic active zone proteins and thereby regulate synaptic vesicle transport and exocytosis to induce synaptic plasticity. Elucidation of these changes in synaptic protein expression provides new insights into the potential role of EE (Figure 5).

## Author contributions

S-YS designed the study, developed the setup, and performed the experiments. MC wrote the manuscript and interpreted the data. JY and ML performed the experiments and analyzed the data. SP wrote the manuscript and conducted data analysis. Y-KS and AB analyzed the data and confirmed the accuracy of the data. J-WP analyzed the data. EP conducted critical revision for the manuscript, and JC and S-RC and wrote the manuscript and conducted study supervision.

### Conflict of interest statement

The authors declare that the research was conducted in the absence of any commercial or financial relationships that could be construed as a potential conflict of interest.
