# Supplementary material for: Environmental Enrichment Upregulates Striatal Synaptic Vesicle-Associated Proteins and Improves Motor Function
Source: Front Neurol. 2018 Jul 16;9:465. doi: 10.3389/fneur.2018.00465 (PMC6054977; doi:10.3389/fneur.2018.00465)
Supplement: Supplementary file 1 [file Table_1.DOC]

**Supplementary Table 1.** Down-regulated proteins by exposure to EE in striatum

| **Accession** | **Description** | **Gene Symbol** | **Fold Change** |
| --- | --- | --- | --- |
| P63094-2 | Isoform Gnas-2 of Guanine nucleotide-binding protein G(s) subunit alpha isoforms short | GNAS | 2.65 |
| Q8CGK7 | Guanine nucleotide-binding protein G(olf) subunit alpha | GNAL | 2.34 |
| P27601 | Guanine nucleotide-binding protein subunit alpha-13 | GNA13 | 2.29 |
| P26883 | Peptidyl-prolyl cis-trans isomerase FKBP1A | FKBP1A | 2.05 |
| Q9QUI0 | Transforming protein RhoA | RHOA | 1.94 |
| E9Q175 | Unconventional myosin-VI | MYO6 | 1.93 |
| F8VPN4 | Protein Agl | AGL | 1.87 |
| Q9CS84-4 | Isoform 4a of Neurexin-1-alpha | NRXN1 | 1.87 |
| B0V2H4 | ATPase, H+ transporting, lysosomal V1 subunit G2 | ATP6V1G2 | 1.86 |
| Q9Z268 | RasGAP-activating-like protein 1 | RASAL1 | 1.86 |
| P52760 | Ribonuclease UK114 | HRSP12 | 1.81 |
| Q62442-2 | Isoform 2 of Vesicle-associated membrane protein 1 | VAMP1 | 1.81 |
| Q8CAY6 | Acetyl-CoA acetyltransferase, cytosolic | ACAT2 | 1.81 |
| Q8BHN3 | Neutral alpha-glucosidase AB | GANAB | 1.77 |
| Q8JZN5 | Acyl-CoA dehydrogenase family member 9, mitochondrial | ACAD9 | 1.77 |
| G3UWG1 | predicted gene 10108; cytochrome c, somatic | GM10108 | 1.75 |
| O88533 | Aromatic-L-amino-acid decarboxylase | DDC | 1.73 |
| Q06185 | ATP synthase subunit e, mitochondrial | ATP5I | 1.73 |
| Q99KJ8 | Dynactin subunit 2 | DCTN2 | 1.73 |
| Q9JI91 | Alpha-actinin-2 | ACTN2 | 1.73 |
| P24288 | Branched-chain-amino-acid aminotransferase, cytosolic | BCAT1 | 1.72 |
| P62748 | Hippocalcin-like protein 1 | HPCAL1 | 1.72 |
| Q91ZR1 | Ras-related protein Rab-4B | RAB4B | 1.72 |
| Q9WUT3 | Ribosomal protein S6 kinase alpha-2 | RPS6KA2 | 1.72 |
| Q80X80 | C2 domain-containing protein 2-like | C2CD2L | 1.71 |
| P97300 | Neuroplastin | NPTN | 1.69 |
| Q8CGC7 | Bifunctional glutamate/proline--tRNA ligase | EPRS | 1.67 |
| Q8VDQ8-2 | Isoform 2 of NAD-dependent protein deacetylase sirtuin-2 | SIRT2 | 1.66 |
| P31938 | Dual specificity mitogen-activated protein kinase kinase 1 | MAP2K1 | 1.66 |
| O88741 | Ganglioside-induced differentiation-associated protein 1 | GDAP1 | 1.66 |
| P31786 | Acyl-CoA-binding protein | DBI | 1.66 |
| Q7TT50 | Serine/threonine-protein kinase MRCK beta | CDC42BPB | 1.66 |
| P05064 | Fructose-bisphosphate aldolase A | ALDOA | 1.64 |
| P08226 | Apolipoprotein E | APOE | 1.63 |
| Q8R570 | Synaptosomal-associated protein 47 | SNAP47 | 1.63 |
| P63005 | Platelet-activating factor acetylhydrolase IB subunit alpha | PAFAH1B1 | 1.62 |
| P35486 | Pyruvate dehydrogenase E1 component subunit alpha, somatic form, mitochondrial | PDHA1 | 1.62 |
| Q60829 | Protein phosphatase 1 regulatory subunit 1B | PPP1R1B | 1.61 |
| P70404 | Isocitrate dehydrogenase [NAD] subunit gamma 1, mitochondrial | IDH3G | 1.59 |
| O35963 | Ras-related protein Rab-33B | RAB33B | 1.59 |
| P70232 | Neural cell adhesion molecule L1-like protein | CHL1 | 1.59 |
| Q64433 | 10 kDa heat shock protein, mitochondrial | HSPE1 | 1.59 |
| Q3UM45 | Protein phosphatase 1 regulatory subunit 7 | PPP1R7 | 1.58 |
| P29758 | Ornithine aminotransferase, mitochondrial | OAT | 1.58 |
| E9Q7Q3 | Tropomyosin alpha-3 chain | TPM3 | 1.57 |
| Q80VM5 | Dipeptidyl aminopeptidase-like protein 6 | DPP6 | 1.57 |
| P62746 | Rho-related GTP-binding protein RhoB | RHOB | 1.57 |
| Q91ZP9 | N-terminal EF-hand calcium-binding protein 2 | NECAB2 | 1.57 |
| Q8R5C5 | Beta-centractin | ACTR1B | 1.56 |
| Q99JY9 | Actin-related protein 3 | ACTR3 | 1.55 |
| P60335 | Poly(rC)-binding protein 1 | PCBP1 | 1.55 |
| P62774 | Myotrophin | MTPN | 1.55 |
| P05063 | Fructose-bisphosphate aldolase C | ALDOC | 1.54 |
| P62761 | Visinin-like protein 1 | VSNL1 | 1.53 |
| P70349 | Histidine triad nucleotide-binding protein 1 | HINT1 | 1.53 |
| Q9WUM4 | Coronin-1C | CORO1C | 1.53 |
| P04370-4 | Isoform 4 of Myelin basic protein | MBP | 1.51 |
| P42669 | Transcriptional activator protein Pur-alpha | PURA | 1.50 |
| Q62465 | Synaptic vesicle membrane protein VAT-1 homolog | VAT1 | 1.50 |
| Q9CQQ7 | ATP synthase subunit b, mitochondrial | ATP5F1 | 1.50 |
| Q9DC69 | NADH dehydrogenase [ubiquinone] 1 alpha subcomplex subunit 9, mitochondrial | NDUFA9 | 1.50 |
| Q80SW1 | Putative adenosylhomocysteinase 2 | AHCYL1 | 1.50 |
