# Supplementary figures and images for: Environmental Enrichment Upregulates Striatal Synaptic Vesicle-Associated Proteins and Improves Motor Function
Source: Front Neurol. 2018 Jul 16;9:465. doi: 10.3389/fneur.2018.00465 (PMC6054977; doi:10.3389/fneur.2018.00465)

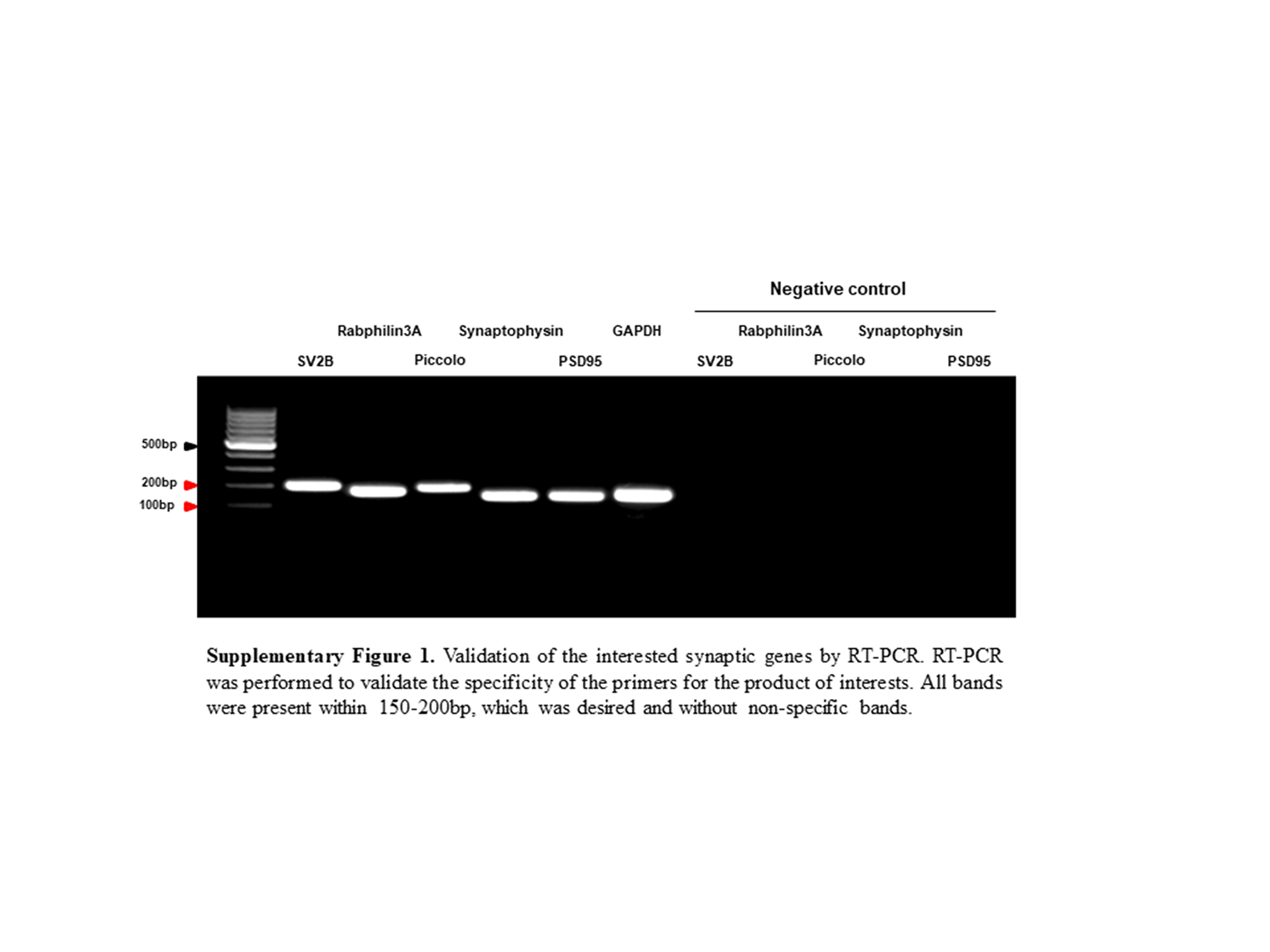

Supplement: Supplementary file 2 [file Image_1.tif]
